# Supplementary material for: Efficient Biocatalytic Synthesis of Dihalogenated Purine Nucleoside Analogues Applying Thermodynamic Calculations
Source: Molecules. 2020 Feb 19;25(4):934. doi: 10.3390/molecules25040934 (PMC7070685; doi:10.3390/molecules25040934)
Supplement: Supplementary file 1 [file molecules-25-00934-s001.pdf]

Supplementary Materials

Table S1. Yields and purity of dihalogenated nucleosides. Yields were determined after each step in the synthesis and purification process. Purity was determined by analytical HPLC. Base concentrations of 5 mM and sugar donor concentrations of 22 to 62.5 mM (Supplementary table 3) were applied in a reaction volume of 50 mL.

**Product**	**Product quantity/ conversion**	**Product quantity/ loss of product per step**		**Total recovery*** **	**Purity**
	**Enzymatic reaction***	**Protein removal + HPLC***	**Purified product****		
**3a**	58 mg/ 83%	47 mg/ 18%	34 mg/28%	49%	98%
**3b**	74 mg/ 92%	51 mg/ 31%	36 mg/ 30%	45%	99%
**3c**	60 mg/ 90%	47 mg/ 21%	32 mg/ 32%	53%	96%
**3d**	59 mg/ 92%	47 mg/ 20%	32 mg/ 32%	54%	99%

* Product quantities were calculated from analytical HPLC data and volumes of the respective solutions ** Weight product. *** Total recovery of the product based on the starting quantity of purine base.

Table S2. NMR characteristics of dihalogenated purine nucleosides produced in the present study.

Compound	NMR spectroscopic data
**3a**<br>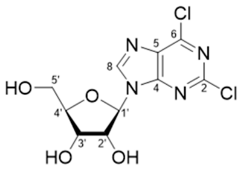<br>$C_{10}H_{10}Cl_2N_4O_4$<br>M = 321.11 g/mol	**^{1}H NMR** (500 MHz, DMSO-d_6): δ = 3.59 (ddd, ^{2}J = 12.0 Hz, ^{3}J = 5.4 Hz, ^{3}J = 4.0 Hz, 1H, H-5′A), 3.71 (ddd, ^{2}J = 12.0 Hz, ^{3}J = 4.6 Hz, ^{3}J = 4.6 Hz, 1H, H-5′B), 3.99 (ddd, ^{3}J = 4.1 Hz, ^{3}J = 3.9 Hz, ^{3}J = 3.9 Hz, 1H, H-4′), 4.18 (ddd, ^{3}J = 4.9 Hz, ^{3}J = 4.9 Hz, ^{3}J = 4.9 Hz, 1H, H-3′), 4.52 (ddd, ^{3}J = 5.3 Hz, ^{3}J = 5.1 Hz, ^{3}J = 5.1 Hz, 1H, H-2′), 5.08 (dd, ^{3}J = 5.4 Hz, ^{3}J = 5.4 Hz, 1H, 5′-OH), 5.25 (d, ^{3}J = 5.4 Hz, 1H, 3′-OH), 5.59 (d, ^{3}J = 5.7 Hz, 1H, 2′-OH), 5.97 (d, ^{3}J = 4.9 Hz, 1H, H-1′), 8.98 (s, 1H, H-8) ppm.<br>**^{13}C{^{1}H} NMR** (126 MHz, DMSO-d_6): δ = 60.7 (C-5′), 69.8 (C-3′), 74.0 (C-2′), 85.7 (C-4′), 88.3 (C-1′), 131.0 (C-5), 146.4 (C-8), 149.8 (C-6), 151.1 (C-2), 153.1 (C-4) ppm.
**3b**<br>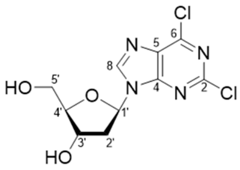<br>$C_{10}H_{10}Cl_2N_4O_3$<br>M = 305.12 g/mol	**^{1}H NMR** (500 MHz, DMSO-d_6): δ = 2.38 (ddd, ^{2}J = 13.5 Hz, ^{3}J = 6.4 Hz, ^{3}J = 4.2 Hz, 1H, H-2′A), 2.71 (ddd, ^{2}J = 13.5 Hz, ^{3}J = 6.2 Hz, ^{3}J = 6.2 Hz, 1H, H-2′B), 3.54 (ddd, ^{2}J = 11.8 Hz, ^{3}J = 5.0 Hz, ^{3}J = 5.0 Hz, 1H, H-5′A), 3.62 (ddd, ^{2}J = 11.8 Hz, ^{3}J = 5.0 Hz, ^{3}J = 5.0 Hz, 1H, H-5′B), 3.89 (ddd, ^{3}J = 4.3 Hz, ^{3}J = 4.3 Hz, ^{3}J = 3.8 Hz, 1H, H-4′), 4.43 (m_c, 1H, H-3′), 4.93 (dd, ^{3}J = 5.5 Hz, ^{3}J = 5.5 Hz, 1H, 5′-OH), 5.36 (d, ^{3}J = 4.4 Hz, 1H, 3′-OH), 6.39 (dd, ^{3}J = 6.4 Hz, ^{3}J = 6.4 Hz, 1H, H-1′), 8.92 (s, 1H, H-8) ppm.<br>**^{13}C{^{1}H} NMR** (126 MHz, DMSO-d_6): δ = 39.4 (C-2′), 61.1 (C-5′), 70.1 (C-3′), 84.3 (C-1′), 88.2 (C-4′), 131.0 (C-5), 146.5 (C-8), 149.7 (C-6), 151.0 (C-2), 152.8 (C-4) ppm.
**3c**<br>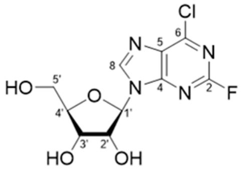<br>$C_{10}H_{10}ClFN_4O_4$<br>M = 304.66 g/mol	**^{1}H NMR** (500 MHz, DMSO-d_6): δ = 3.59 (ddd, ^{2}J = 12.0 Hz, ^{3}J = 5.4 Hz, ^{3}J = 4.0 Hz, 1H, H-5′A), 3.70 (ddd, ^{2}J = 12.0 Hz, ^{3}J = 5.1 Hz, ^{3}J = 4.2 Hz, 1H, H-5′B), 3.99 (ddd, ^{3}J = 4.1 Hz, ^{3}J = 4.0 Hz, ^{3}J = 4.0 Hz, 1H, H-4′), 4.18 (ddd, ^{3}J = 4.9 Hz, ^{3}J = 4.9 Hz, ^{3}J = 4.9 Hz, 1H, H-3′), 4.52 (ddd, ^{3}J = 5.3 Hz, ^{3}J = 5.1 Hz, ^{3}J = 5.1 Hz, 1H, H-2′), 5.07 (dd, ^{3}J = 5.4 Hz, ^{3}J = 5.4 Hz, 1H, 5′-OH), 5.25 (d, ^{3}J = 5.4 Hz, 1H, 3′-OH), 5.59 (d, ^{3}J = 5.7 Hz, 1H, 2′-OH), 5.94 (d, ^{3}J = 4.9 Hz, 1H, H-1′), 8.95 (s, 1H, H-8) ppm.<br>**^{13}C{^{1}H} NMR** (126 MHz, DMSO-d_6): δ = 60.8 (C-5′), 69.8 (C-3′), 74.0 (C-2′), 85.6 (C-4′), 88.3 (C-1′), 130.5 (d, $^{4}J_{C,F}$ = 4.9 Hz, C-5), 146.4 (C-8), 150.6 (d, $^{3}J_{C,F}$ = 18.5 Hz, C-6), 153.5 (d, $^{3}J_{C,F}$ = 17.4 Hz, C-4), 156.1 (d, $^{1}J_{C,F}$ = 214.1 Hz, C-2) ppm.<br>**^{19}F{^{1}H} NMR** (471 MHz, DMSO-d_6): δ = –51.7 ppm.
**3d**<br>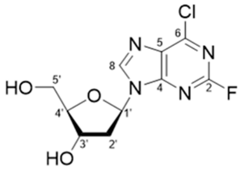<br>$C_{10}H_{10}ClFN_4O_3$<br>M = 288.66 g/mol	**^{1}H NMR** (500 MHz, DMSO-d_6): δ = 2.37 (ddd, ^{2}J = 13.5 Hz, ^{3}J = 6.4 Hz, ^{3}J = 4.2 Hz, 1H, H-2′A), 2.71 (ddd, ^{2}J = 13.5 Hz, ^{3}J = 6.3 Hz, ^{3}J = 6.3 Hz, 1H, H-2′B), 3.53 (dd, ^{2}J = 11.8 Hz, ^{3}J = 4.5 Hz, 1H, H-5′A), 3.61 (dd, ^{2}J = 11.8 Hz, ^{3}J = 4.5 Hz, 1H, H-5′B), 3.89 (ddd, ^{3}J = 4.3 Hz, ^{3}J = 4.3 Hz, ^{3}J = 3.7 Hz, 1H, H-4′), 4.43 (ddd, ^{3}J = 5.7 Hz, ^{3}J = 3.9 Hz, ^{3}J = 3.9 Hz, 1H, H-3′), 4.94 (br s, 1H, 5′-OH), 5.37 (br s, 1H, 3′-OH), 6.36 (dd, ^{3}J = 6.4 Hz, ^{3}J = 6.4 Hz, 1H, H-1′), 8.89 (s, 1H, H-8) ppm.<br>**^{13}C{^{1}H} NMR** (126 MHz, DMSO-d_6): δ = 39.3 (C-2′), 61.1 (C-5′), 70.1 (C-3′), 84.3 (C-1′), 88.1 (C-4′), 130.5 (d, $^{4}J_{C,F}$ = 5.0 Hz, C-5), 146.5 (d, $^{5}J_{C,F}$ = 2.9 Hz, C-8), 150.6 (d, $^{3}J_{C,F}$ = 18.5 Hz, C-6), 153.5 (d, $^{3}J_{C,F}$ = 17.4 Hz, C-4), 156.1 (d, $^{1}J_{C,F}$ = 214.2 Hz, C-2) ppm.<br>**^{19}F{^{1}H} NMR** (471 MHz, DMSO-d_6): δ = –51.7 ppm.

Table S3. Conditions used for the synthesis of dihalogenated purine nucleosides with a product yield of 90% or 95%. Reaction temperature was 40°C.

**Product**	**3a**	**3b**	**3c**	**3d**
Sugar donor 90%/95% product yield	22.5/45 mM **1a**	30.5/62.5 mM **1b**	22/44 mM **1a**	27/55 mM **1b**
Base	5 mM **2a**	5 mM **2a**	5 mM **2b**	5 mM **2b**
Enzymes/ final enzyme conc.	PyNP 02/ 0.1 mg ml^{-1}, PNP 02/ 0.1 mg mL^{-1}			

Table S4. HPLC methods applied for the purification of the dihalogenated nucleoside analogues. A flow rate 21.24 ml min^{-1} was employed. Acetonitrile (ACN) and water were applied as solvents.

Target compound	Injected sample volume [mL]	HPLC gradient
**3a**, **3c**	4	Initial 3% ACN; linear gradient to 22% ACN in 12.5 min; the initial condition (3% ACN) was restored within 0.5 min and kept for 1 min
**3b**	2	Initial 3% ACN; linear gradient to 25% ACN in 15 min; the initial condition (3% ACN) was restored within 1 min and kept for 2 min
**3d**	5	Initial 3% ACN; linear gradient to 25% ACN in 10 min; the initial condition (3% ACN) was restored within 1 min and kept for 1.5 min

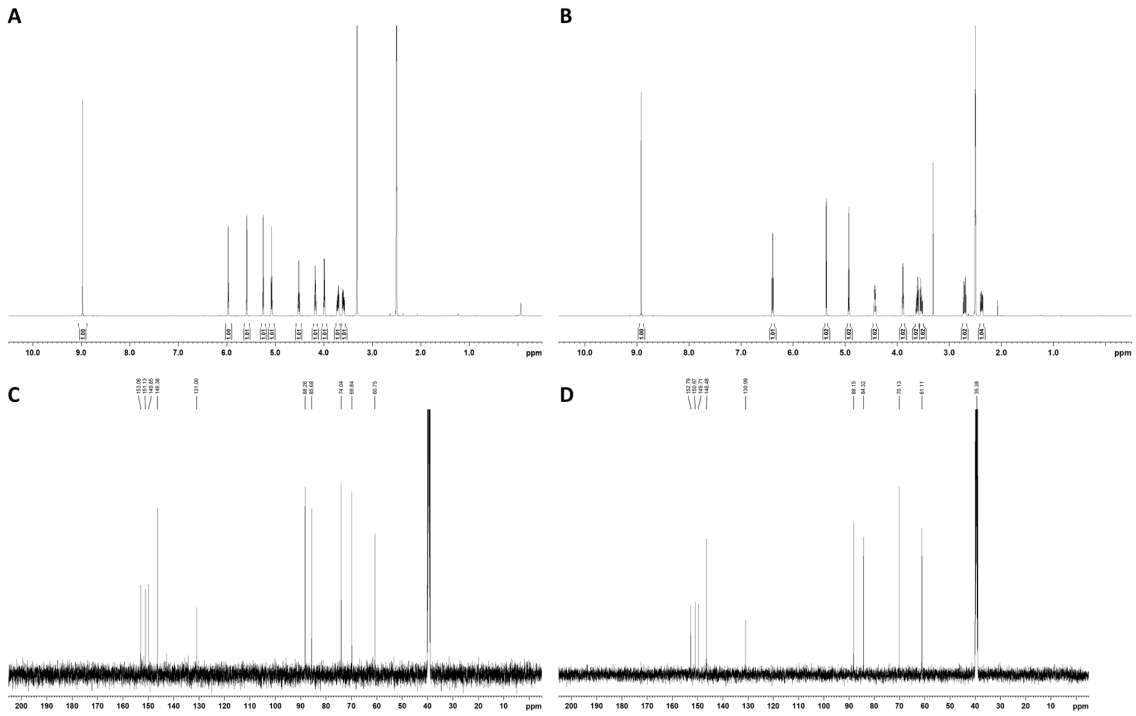

Figure S1. NMR spectra for DCP-R (A, C) and DCP-dR (B, D). A, B: ^{1}H NMR (500 MHz, DMSO-d_6), C, D: $^{13}C\{^{1}H\}$ NMR (126 MHz, DMSO-d_6)

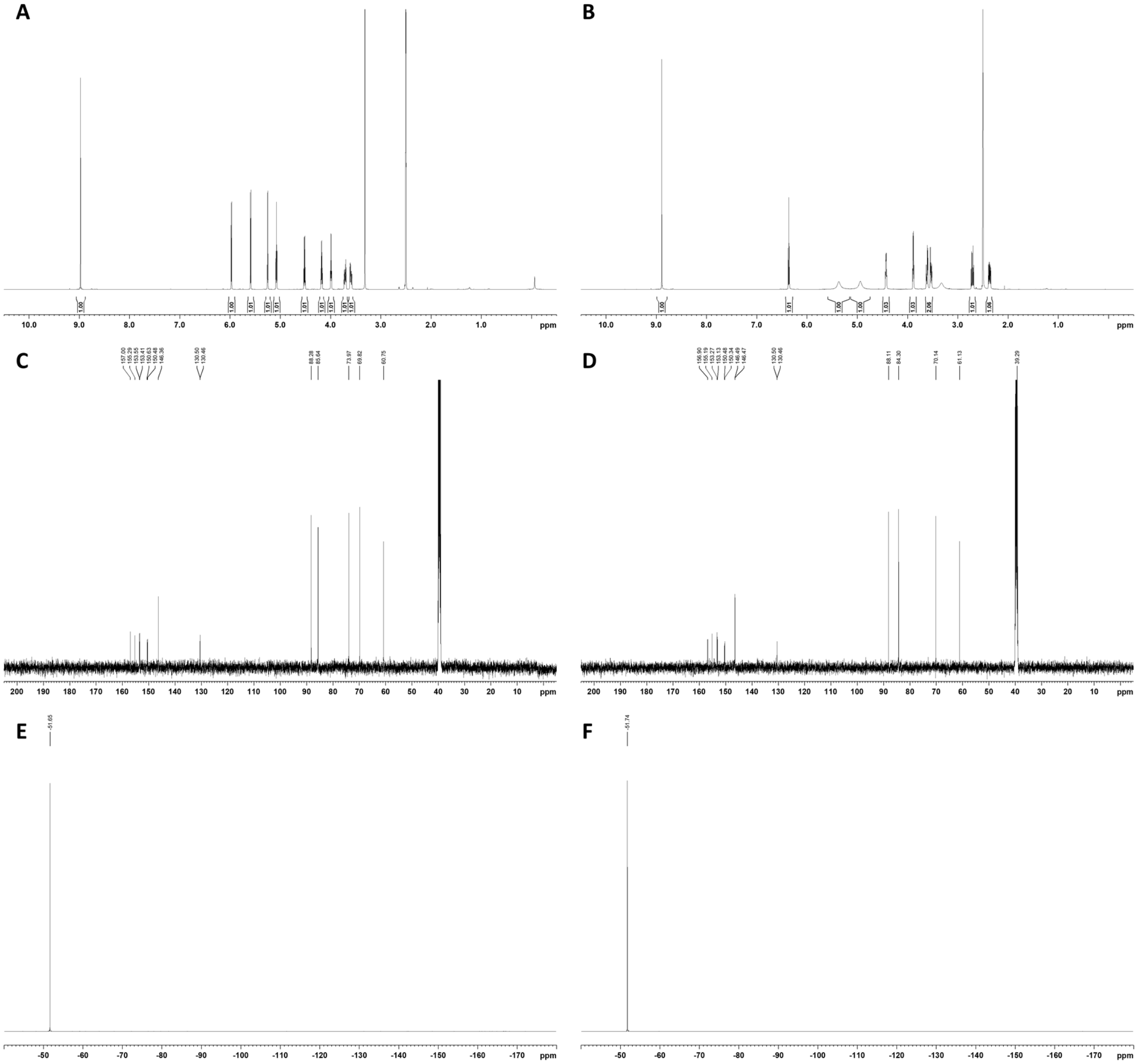

Figure S2. NMR spectra for CFP-R (A, C, E) and CFP-dR (B, D, F). A, B: ^{1}H NMR (500 MHz, DMSO-d$_6$); C, D: $^{13}C\{^{1}H\}$ NMR (126 MHz, DMSO-d$_6$); E,F: $^{19}F\{^{1}H\}$ NMR (471 MHz, DMSO-d_6)
